# Brown fat detection by infrared thermography—An invaluable research methodology with noteworthy uncertainties confirmed by a mathematical proof

**DOI:** 10.1002/edm2.378

**Published:** 2022-10-31

**Authors:** Melvin K. S. Leow

**Affiliations:** ^1^ Department of Human Development Singapore Institute for Clinical Sciences, A*STAR Singapore City Singapore; ^2^ Lee Kong Chian School of Medicine Singapore City Singapore; ^3^ Cardiovascular and Metabolic Disorders Program Duke‐NUS Medical School Singapore City Singapore; ^4^ Department of Endocrinology Tan Tock Seng Hospital Singapore City Singapore

**Keywords:** brown adipose tissue, detection accuracy, fat segmentation, imaging, infrared thermography, precision

## Abstract

Brown adipose tissue (BAT) represents a pivotal scientific renaissance worthy as a strategy for obesity and diabetes since its re‐discovery in adults over a decade ago. Equally compelling is the adoption of infrared thermography (IRT) in recent times as a precise and viable alternative methodology over the ‘gold standard’ PET‐CT scan, given constraints of the latter's high ionizing radiation doses and costs. Unravelling BAT metabolic physiology in live humans has been challenging until recent rigorous validation of IRT against PET. Nevertheless, IRT remains a nascent technique with pitfalls unbeknownst to many researchers. Factors impacting its accuracy merit an in‐depth scientific scrutiny. This article discusses the strengths and pitfalls of IRT as an emergent BAT detection technique and provides a mathematical proof of its limitations that BAT researchers should be cognizant of. Understanding these limitations of IRT can prompt extra efforts to control these uncertainties with greater rigour. In conclusion, this warrants further investigations of improving IRT quality via advanced auto‐segmentation, powerful image processing of thermograms and protocol standardization along the lines of BARCIST 1.0 to minimize errors and enhance the confidence of the global BAT research community in IRT as a robust and reliable BAT research tool.

## Graphical abstract

Infrared detection by thermal camera obeys Stefan's law of radiative heat transfer. This associated uncertainty in detection is estimated by the formula of propagation of error.

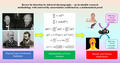

## 1 INTRODUCTION AND BACKGROUND

Brown adipose tissue (BAT) represents a pivotal scientific renaissance worthy as a strategy for obesity and diabetes since its re‐discovery in adults over a decade ago.^1^, ^2^, ^3^ Equally compelling is the adoption of infrared thermography (IRT) in recent times as a precise and viable alternative methodology over the ‘gold standard’ PET‐CT scan, given constraints of the latter's high ionizing radiation doses and costs. Unravelling BAT metabolic physiology in live humans has been challenging until recent rigorous validation of IRT against PET.^4^, ^5^, ^6^, ^7^ Nevertheless, IRT remains a nascent technique with pitfalls unbeknownst to many researchers. Adopting the mindset of a mathematician and theoretical biologist, I shall illustrate the key factors impacting its accuracy which merit an in‐depth scientific scrutiny via a rigorous mathematical proof.

## 2 QUANTUM THERMODYNAMICS OF PHOTON–PHONON COUPLING

To better comprehend IRT and its limitations, a basic knowledge of quantum theory of electromagnetic (EM) radiation helps. The Planck‐Einstein's energy (ℇ) equation states:

(1) ℇ=hν

where *h* (the Planck's constant) = 6.626 × 10^−34^ Js, and ν refers to frequency.^8^ Infrared corresponds to a frequency range of 300 GHz to 400 THz, or wavelengths between 780 nm and 1 mm. Given the reality of the inseparable wave‐particle duality, EM waves have simultaneous properties in the form of momentum‐possessing massless photons. This is best enunciated by de Broglie's law:

(2) λ=h/p

where *h* is the Planck's constant, *λ* is the wavelength of the EM rays, and *p* is the momentum.^8^ By first principles of quantum mechanics, the Schroedinger's equation links the wave function *ψ* of particles to their total energy via the Hamiltonian operator *H* such that:

(3) $$\frac{\mathrm{ih}}{2\pi }\frac{\partial }{\partial t}\psi =\mathrm{H\psi}=-\frac{h^{2}}{8\pi^{2}m}\nabla^{2}\;\psi +V\left(x\right)\psi$$

whereby the imaginary number *i* = $\sqrt{-1}$, *π* = 3.142, *h* is the Planck's constant, and $\nabla^{2}$ is the Laplacian operator commonly expressed in 3D as $\frac{\partial^{2}}{\partial x^{2}}+\frac{\partial^{2}}{\partial y^{2}}+\frac{\partial^{2}}{\partial z^{2}}$ in Cartesian or also as $\frac{1}{r^{2}}\frac{\partial }{\partial r}\left(r^{2}\frac{\partial }{\partial r}\right)+\frac{1}{r^{2}\sin ϴ}\frac{\partial }{\partial ϴ}\left(\sin ϴ\frac{\partial }{\partial ϴ}\right)+\frac{1}{r^{2}\sin^{2}ϴ}\frac{\partial^{2}}{\partial \emptyset^{2}}$ in polar coordinates frames of reference.

Equation (3) expresses by Hamiltonian formalism the canonical operator *H* which is a geometric transformation linking the energy to a wave function in momentum phase space, such that:

(4) $$H=-\frac{h^{2}}{8\pi^{2}m}\frac{\partial^{2}}{\partial x^{2}}+V\left(x\right)$$

where the first term represents the kinetic energy operator while *V(x)* represents the potential energy.^8^ The eigenvalues of the Hamiltonian are all possible outcomes of the total ‘quantized’ energy.

A common misconception is that infrared radiation is ‘heat energy’. Infrared photons are not “hot” and neither do they possess heat per se. Rather, when infrared quanta strike matter, their momentum is transferred to atoms due to precise coupling interactions between possible Hamiltonian excitation energy levels of molecules with infrared photons acting as harmonic oscillators which transfer vibrational energy for heat transport.^9^ Heat is mechanical vibration at the atomic level, and a thermal phonon is a quasiparticle of a definite discrete quantum of vibrational mechanical energy.^10^ This photon–phonon coupling only occurs with distinct EM frequencies, and infrared photons are quantized perfectly to resonate with phonons within matter and vice versa, allowing spontaneous absorption and emission. Thermal phonons from matter can thus generate infrared photons detectable by sensors of an infrared camera.^10^

## 3 OPTICS OF INFRARED THERMOGRAPHY

Most researchers applying IRT for the detection of heat emanating from the skin surface overlying activated BAT within the region of interest (ROI) only consider temperature as the key data readout. Not appreciating the physics of IRT obscures its potential weaknesses when utilized in young babies or small animals. All modern infrared cameras work via lenses made not of glass but of germanium or zinc selenide which are transparent to infrared radiation. The transmitted photons are focused onto a sensor array of photoelectric pixels that generate electrical energy upon interaction with these photons.^11^ The subsequent electronic signal is then sent to a computerized processor that renders a false colour map of different temperature values based on the original energy of the infrared photons.^11^

Heat detection using a thermal imager depends on temperature but not on distance according to Planck's radiation law, which implies that the variable focal lengths of 40–100 cm between the imager and the young babies often used by BAT researchers are inconsequential in terms of errors.^12^, ^13^ By contrast, because the circumscribed BAT ROI based on thermograms of published studies^12^, ^13^, ^14^ have an estimated dimension in the order of approximately 50 cm^2^ given the physical sizes of neonates and small children, this presents an inherent weakness many researchers had not foreseen. The following calculus‐derived statistical error analysis elegantly illustrates this issue by propagating the uncertainties from two key variables, chiefly the ROI area (*A*) and skin temperature (*T*).

## 4 MATHEMATICAL PROOF OF IRT ERRORS

To understand the errors emerging from IRT, a formal uncertainty analysis of the Stefan‐Boltzmann equation,^15^, ^16^ a fourth power law derivable by integrating Planck's radiation law over all wavelengths governing heat transmission through space is shown:

(5) $$E=\mathrm{\sigma \epsilon A}T^{4}$$

where 𝜎 and ϵ are Stefan's constant (= 5.6 × 10^−8^ W/m^2^ K^4^) and emissivity respectively, and *A* denotes area of skin ROI overlying BAT, *T* is the temperature and *E* is the heat power as captured by IRT sensors.

Since *A* and *T* are independent variables, the rates of change of *E* with respect to *A* and *T* are the respective partial derivatives:

(6) $$\;\frac{\partial E}{\partial A}=\sigma \epsilon T^{4}$$

(7) $$\frac{\partial E}{\partial T}=\mathrm{\sigma \epsilon A}\left(4T^{3}\right)$$

Applying the formula for the propagation of errors based on a theorem of Cramér to compute total error Δ*E* as variance^17^ in terms of ∆A (error in *A*) and ∆T (error in *T*), this reduces to:

(8) $${\left(∆E\right)}^{2}={\left(\frac{\partial E}{\partial A}\right)}^{2}{\left(∆A\right)}^{2}+{\left(\frac{\partial E}{\partial T}\right)}^{2}{\left(∆T\right)}^{2}$$

since the covariance is zero (⸪ *A* and *T* are independent) and the term corresponding to the contribution by this component of equation (8), namely 2 $\left(\frac{\partial E}{\partial A}\right)\left(\frac{\partial E}{\partial T}\right)\frac{\sum_{i=1}^{n}\left(∆\mathrm{Ai}\right)\left(∆\mathrm{Ti}\right)}{n}$ vanishes.

Hence, substituting Equations (6) and (7) into (8) yields the variance:

(9) $${\left(∆E\right)}^{2}=\sigma^{2}\epsilon^{2}\left[T^{8}{\left(∆A\right)}^{2}+16A^{2\;}T^{6}{\left(∆T\right)}^{2}\right]$$

From the physical locations and reported sizes of BAT depots in humans,^2^, ^18^, ^19^ the area (*A*) of skin overlying BAT is approximately 1–400 cm^2^, whereas temperature change in *T* as detected with BAT activation is typically approximately 0.2–1.5 K.^6^, ^7^, ^20^, ^21^

Depending on the angle and parallax, as well as the deformation of the skin due to movement and positioning, the ROI view captured by the thermal camera is operator‐dependent and can vary in magnitude to the order of 5–15 cm^2^. The thermal sensitivity of IRT cameras typically ranges from 0.005 K to 0.05 K. To simplify the computations, we assume the average errors in *A* and *T* as:

$$\;\Delta A=10\;{\mathrm{cm}}^{2}$$

∆T=0.01K

From equation (9), in order to understand which error component is greater, we compare the two terms within the parentheses and define each term as $\varepsilon_{A}\;\text{and}\;\varepsilon_{T}$ for the error contribution by area and temperature, respectively:

(10) $$\varepsilon_{A}=T^{8}{\left(∆A\right)}^{2}$$

(11) $$\varepsilon_{T}=16A^{2\;}T^{6}{\left(∆T\right)}^{2}$$

Therefore, we evaluate the error ratio *ρ* as:

(12) $$\rho =\frac{\varepsilon_{A}}{\varepsilon_{T}}=\frac{T^{8}{\left(∆A\right)}^{2}}{16{\left(0.01\right)}^{2}T^{6}A^{2}}=\frac{T^{2}{\left(∆A\right)}^{2}}{1.6\times 10^{-3}\times A^{2}}=625T^{2}{\left(\frac{∆A}{A}\right)}^{2}\;$$

Since magnitude of temperature rise with BAT activation almost certainly fall within this range of approximately 0.2 ≤ *T* ≤ 1.5, we have:

$$25\;{\left(\frac{∆A}{A}\right)}^{2}\leq \rho \leq 1406\;{\left(\frac{∆A}{A}\right)}^{2}$$

Substituting Δ*A* = 10 cm^2^, this error analysis leads to the conclusion that:

- for an area *A* ≥ 375 cm^2^, the noise contribution from temperature measurement errors is greater, whereas the error source from ROI variations is greater if *A* ≤ 50 cm^2^.

As the area of the neonates' and infants' BAT ROI may well be <50 cm^2^, this implies that the IRT in this population may suffer from significant inaccuracies.

To further validate the argument, we can perform an independent uncertainty analysis by examining conditional variance contributions, $\mathrm{Var}\;\left(E|A\right)$ and $\mathrm{Var}\;\left(E|T\right)$, from which the source of errors contributed by *A* and *T* may be compared.

Let us denote the errors in *A* and *T* by ε_*A*_ and $\varepsilon_{T},$ respectively, and assume both errors to follow the Gaussian distribution:

(13) $$\varepsilon_{A}\sim N\left(0,{\sigma_{\varepsilon_{A}}}^{2}\right)$$

(14) $$\varepsilon_{T}\sim N\;\left(0,{\sigma_{\varepsilon_{T}}}^{2}\right)$$

Thus, variance contributions at any paired coordinate (*A*,*T*), each having normal distributions ε_*A*_ and ε_*T*_, can be compared as a logarithmic function of the ratio of their conditional variance:

(15) $$\rho \left(A,T\right)=\log\left(\frac{\mathrm{Var}\left(E|A\right)}{\mathrm{Var}\left(E|T\right)}\right)=\log\left(\frac{A^{2}\mathrm{Var}\left(T^{4}\right)}{T^{8}\mathrm{Var}\left(A\right)}\right)$$

If $\rho \left(A,T\right)>0,\text{then}\;\frac{\mathrm{Var}\left(E|A\right)}{\mathrm{Var}\left(E|T\right)}>1$, which then implies that the error contribution from *T* is greater than *A* under such circumstances. Similarly, if $\rho \left(A,T\right)<0,\text{then}\;\frac{\mathrm{Var}\left(E|A\right)}{\mathrm{Var}\left(E|T\right)}<1,$ in which case the error contribution by *A* exceeds that arising from measurements of *T*.

The estimation of Var(*A*) and Var($T^{4})$ can be computed via a Monte Carlo simulation to evaluate $\rho \left(A,T\right)$ over the entire sample space of (*A*,*T*) $\in \left[1400\right]\;{\mathrm{cm}}^{2}\times \left[0.1,1.5\right]\;K.$

This alternative approach to uncertainty analysis can provide realistic estimates of the area cutoffs that influence the model's overall uncertainty. Such an in silico experimental simulation is shown by Table 1 from our own study.^22^ Delineating the negative (green cells) from the positive (red cells) values shows that area poses a greater determinant of error when *A* < 50 cm^2^, whereas temperature predominates as a greater contributor of error when *A* > 200 cm^2^. These cutoffs are similar to the orders of magnitude as those derived analytically above.

**TABLE 1:** Monte Carlo simulation results showing relative contributions of errors by variables in the Stefan‐Boltzmann equation (Adapted from Tay SH et al. [2020] with permission from *Physiological Research* journal)

		*T*														
		0.1	0.2	0.3	0.4	0.5	0.6	0.7	0.8	0.9	1	1.1	1.2	1.3	1.4	1.5
*A*	1	−5.01724	−6.42937	−7.25368	−7.81986	−8.27235	−8.63736	−8.9351	−9.22196	−9.4377	−9.65301	−9.84648	−10.0116	−10.1819	−10.3367	−10.462
	20	0.957888	−0.43768	−1.25819	−1.8207	−2.2816	−2.64628	−2.95247	−3.22193	−3.46627	−3.66063	−3.85379	−4.0371	−4.20258	−4.34348	−4.475
	40	2.351156	0.943114	0.131362	−0.4496	−0.89846	−1.25991	−1.56936	−1.83106	−2.05789	−2.28059	−2.4713	−2.64136	−2.79517	−2.94782	−3.09159
	60	3.158902	1.751267	0.947505	0.357812	−0.08623	−0.46087	−0.74364	−1.01795	−1.24496	−1.46695	−1.65938	−1.83726	−1.9957	−2.15009	−2.28061
	80	3.74958	2.331787	1.519434	0.94036	0.493734	0.137693	−0.17737	−0.43057	−0.67489	−0.88804	−1.08863	−1.25741	−1.41626	−1.56987	−1.70904
	100	4.192137	2.777924	1.96224	1.385459	0.942219	0.57915	0.262283	0.00729	−0.24158	−0.44629	−0.62915	−0.80945	−0.97721	−1.11308	−1.25206
	120	4.547355	3.154345	2.323958	1.750481	1.305048	0.944567	0.641611	0.37022	0.134439	−0.08918	−0.27284	−0.44561	−0.615	−0.75513	−0.89563
	140	4.862527	3.457785	2.623848	2.059246	1.611413	1.258439	0.942213	0.669412	0.436678	0.223238	0.036067	−0.14886	−0.30705	−0.46228	−0.58633
	160	5.11517	3.716954	2.913853	2.331688	1.892016	1.510915	1.200088	0.951912	0.710647	0.491474	0.30425	0.12616	−0.02795	−0.17876	−0.3169
	180	5.362825	3.955759	3.143629	2.568967	2.106529	1.753154	1.442427	1.17402	0.939133	0.716625	0.541782	0.36033	0.202714	0.062414	−0.09
	200	5.56994	4.165467	3.357518	2.785091	2.332748	1.958435	1.649685	1.39618	1.156669	0.937405	0.753841	0.567403	0.401919	0.266784	0.123857
	220	5.757021	4.358699	3.541999	2.973492	2.51768	2.154727	1.838144	1.57418	1.333037	1.116673	0.949202	0.767241	0.601727	0.455477	0.319341
	240	5.936099	4.522318	3.720827	3.144228	2.68923	2.332331	2.025288	1.75976	1.518556	1.307297	1.117491	0.947447	0.782094	0.632304	0.501369
	260	6.085678	4.689268	3.868927	3.286489	2.851338	2.478483	2.180399	1.911968	1.675603	1.464412	1.270303	1.109232	0.930066	0.79539	0.661866
	280	6.24728	4.832832	4.035036	3.446165	3.001775	2.633291	2.338673	2.055185	1.816484	1.605168	1.426008	1.241855	1.088315	0.93859	0.800406
	300	6.377543	4.975629	4.167501	3.58312	3.14232	2.767282	2.464705	2.20266	1.972249	1.756983	1.553982	1.392862	1.220467	1.070983	0.937106
	320	6.508326	5.104898	4.288326	3.706786	3.273462	2.894967	2.603795	2.326495	2.095157	1.886793	1.686211	1.514672	1.350662	1.200117	1.064082
	340	6.63892	5.225362	4.41548	3.82533	3.403986	3.039095	2.713483	2.452661	2.223989	2.005738	1.804398	1.627522	1.478585	1.325599	1.188154
	360	6.753761	5.343352	4.527108	3.951022	3.512419	3.130149	2.82677	2.567453	2.328343	2.124803	1.914614	1.759319	1.598573	1.449455	1.294263
	380	6.863783	5.46112	4.629714	4.06401	3.609608	3.237098	2.937492	2.671313	2.439282	2.233642	2.024782	1.8604	1.700445	1.545163	1.41517
	400	6.970084	5.552408	4.743284	4.15854	3.724521	3.351161	3.045501	2.769181	2.543245	2.325123	2.131883	1.949306	1.78948	1.657991	1.514338

*Note*: Each cell represents $\rho \left(A,T\right)=\log\left(\frac{\mathrm{Var}\left(E|A\right)}{\mathrm{Var}\left(E|T\right)}\right)=\log\left(\frac{A^{2}\mathrm{Var}\left(T^{4}\right)}{T^{8}\mathrm{Var}\left(A\right)}\right)$. For each pair (*A*,*T*), the variance of *T* is greater than that of *A* if the value of the cell >0, whereas the variance of *A* is greater than that of *T* if the value of the cell <0. For ease of visualization, cells >0 are coloured red while cells <0 are coloured green. *A*, area of ROI overlying the BAT depot; *T*, temperature increase in the ROI with BAT activation.

## 5 SUMMARY AND CONCLUDING REMARKS

Taken together, this treatise unravels an imprecision of IRT for ROI < 50 cm^2^. For adults, BAT detection by IRT of the lateral neck and supraclavicular regions which seldom exceed 200 cm^2^ would be relatively accurate by contrast. Understanding these limitations of IRT can prompt extra efforts to control these uncertainties with greater rigour. In conclusion, this warrants further investigations of improving IRT quality via advanced auto‐segmentation, powerful image processing of thermograms and protocol standardization along the lines of BARCIST 1.0 to minimize errors and enhance the confidence of the global BAT research community in IRT as a robust and reliable BAT research tool.^23^

## AUTHOR CONTRIBUTIONS

**Melvin Leow:** Conceptualization (lead); formal analysis (lead); methodology (lead); resources (lead); software (equal); validation (equal); writing – original draft (lead); writing – review and editing (lead).

## CONFLICT OF INTEREST

The author declares no competing interests.

## FUNDING INFORMATION

This work was not supported by any grant. The Singapore Institute for Clinical Sciences, A*STAR, funded the publication fee.

## Data Availability

Not applicable as no experimental data was used in the mathematical analysis.
